# Cofactor engineering for improved production of 2,4-dihydroxybutyric acid via the synthetic homoserine pathway

**DOI:** 10.3389/fbioe.2025.1504785

**Published:** 2025-02-20

**Authors:** Nadine Ihle, Laura Grüßner, Ceren Alkim, T. A. Stefanie Nguyen, Thomas Walther, Cláudio J. R. Frazão

**Affiliations:** ^1^ Chair of Bioprocess Engineering, Institute of Natural Materials Technology, TU Dresden, Dresden, Germany; ^2^ Toulouse Biotechnology Institute, UMR INSA-CNRS5504 and UMR INSA-INRAE 792, Toulouse, France

**Keywords:** enzyme engineering, strain engineering, cofactor specificity, synthetic metabolic pathway, 2,4-dihydroxybutyric acid, homoserine, *Escherichia coli*

## Abstract

(L)-2,4-dihydroxybutyrate (DHB) is a versatile compound that can serve as a precursor for the synthesis of the methionine analog 2-hydroxy-4-(methylthio)butyrate and new advanced polymers. We previously implemented in *Escherichia coli* an artificial biosynthetic pathway for the aerobic production of DHB from glucose, which relies on the deamination of (L)-homoserine followed by the reduction of 2-oxo-4-hydroxybutyrate (OHB) and yields DHB by an enzyme-bearing NADH-dependent OHB reductase activity. Under aerobic conditions, using NADPH as a cofactor is more favorable for reduction processes. We report the construction of an NADPH-dependent OHB reductase and increased intracellular NADPH supply by metabolic engineering to improve DHB production. Key cofactor discriminating positions were identified in the previously engineered NADH-dependent OHB reductase (*E. coli* malate dehydrogenase I12V:R81A:M85Q:D86S:G179D) and tested by mutational scanning. The two point mutations D34G:I35R were found to increase the specificity for NADPH by more than three orders of magnitude. Using the new OHB reductase enzyme, replacing the homoserine transaminase with the improved variant Ec.AlaC A142P:Y275D and increasing the NADPH supply by overexpressing the *pntAB* gene encoding the membrane-bound transhydrogenase yielded a strain that produced DHB from glucose at a yield of 0.25 mol_DHB_ mol_Glucose_^−1^ in shake-flask experiments, which corresponds to a 50% increase compared to previous producer strains. Upon 24 h of batch cultivation of the most advanced DHB producer strain constructed in this work, a volumetric productivity of 0.83 mmol_DHB_ L^−1^ h^−1^ was reached.

## 1 Introduction

(L)-2,4-dihydroxybutyrate (DHB) is a versatile compound of growing industrial relevance, as it can serve as a precursor for the chemical production of the methionine analog 2-hydroxy-4-(methylthio)butyrate (HMTB) used in animal nutrition (Walther et al., 2017a) or as a building block for new advanced biopolymers (François, 2023). Furthermore, DHB can serve as a precursor for the synthesis of 1,3-propanediol (Frazão et al., 2019) or 1,2,4-butanetriol (Li et al., 2014). Although the occurrence of DHB at trace levels in patients with succinic semialdehyde dehydrogenase deficiency has been described previously (Shinka et al., 2002), there is no annotated natural metabolic pathway for its biosynthesis.

**FIGURE 1:**
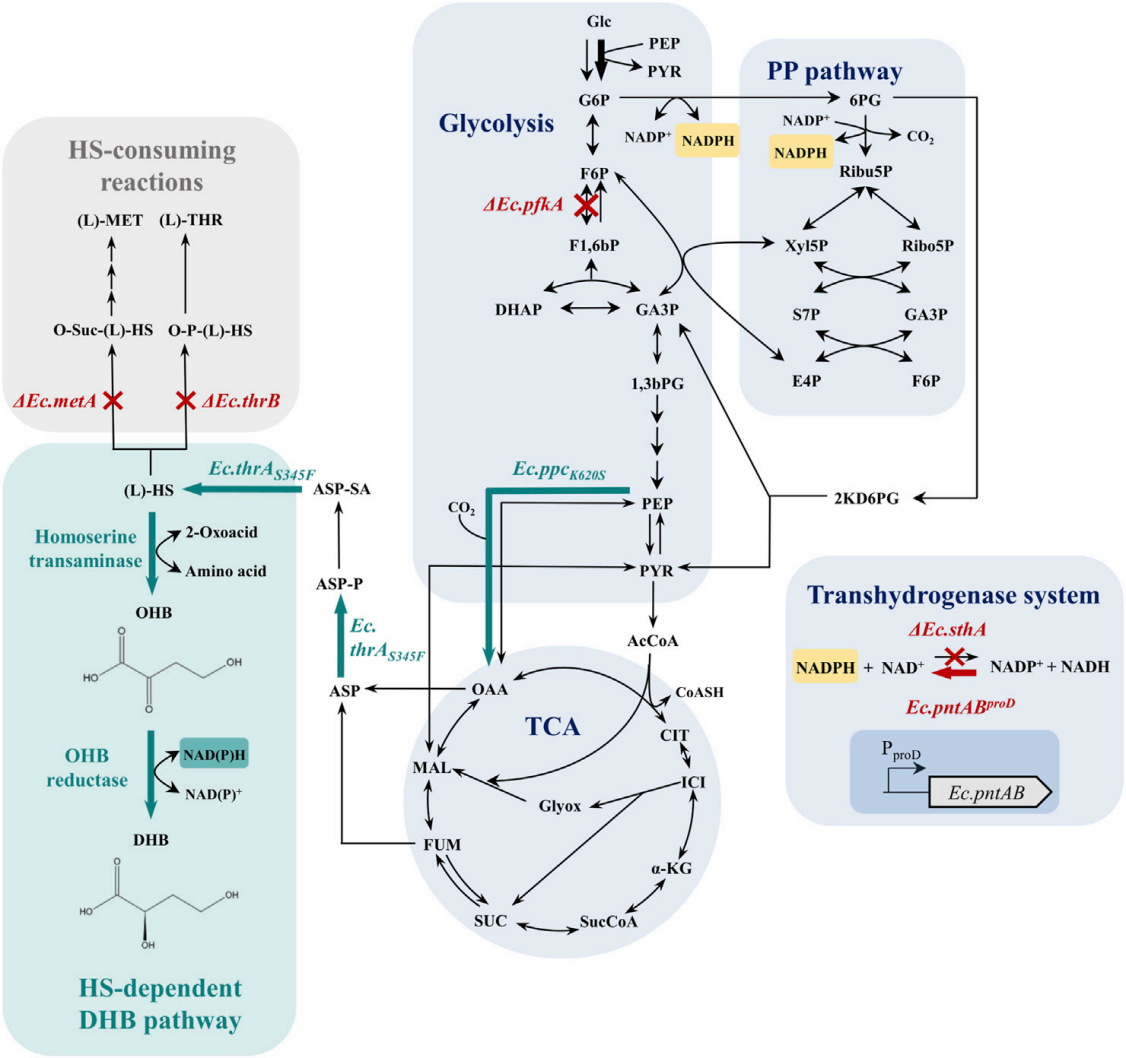
Aerobic synthesis of (L)-2,4-dihydroxybutyric acid (DHB) from glucose via the artificial homoserine pathway (bluish-green) and applied strategies for increased NADPH availability in *Escherichia coli*. Abbreviations: 1,3bPG, 1,3-bisphospho-glycerate; AcCoA, acetyl-CoA; ASP, aspartate; ASP-P, aspartyl phosphate; ASP-SA, aspartate semialdehyde; CIT, citrate; DHAP, dihydroxyacetone phosphate; F1,6bP, fructose-1,6-bisphosphate; F6P, fructose-6-phosphate; FUM, fumarate; G3P, glyceraldehyde-3-phosphate; G6P, glucose-6-phosphate; Glc, glucose; ICI, isocitrate; (L)-DHB, (L)-2,4-dihydroxybutyrate; (L)-HS, (L)-homoserine; (L)-MET, (L)-methionine; (L)-THR, (L)-threonine; MAL, malate; OAA, oxaloacetate; OHB, 2-oxo-4-hydroxybutyrate; O-P-(L)-HS, *O*-phospho-L-homoserine; O-Suc-(L)-HS, *O*-succinyl-(L)-homoserine; PEP, phosphoenolpyruvate; PP pathway, pentose phosphate pathway; PYR, pyruvate; SUC, succinate; SucCoA, succinyl-CoA; TCA, tricarboxylic acid cycle; α-KG, α-ketoglutarate; Gene names are written in italics. *Ec.pfkA*–gene encoding 6-phosphofructokinase; *Ec.sthA*–gene encoding soluble pyridine nucleotide transhydrogenase; *Ec.pntAB*–gene encoding membrane-bound pyridine nucleotide transhydrogenase; *Ec.ppc*_*K620S*_–gene encoding aspartate/malate insensitive phosphoenolpyruvate carboxylase mutant (Walther et al., 2017a); *Ec.thrA*_*S345F*_–gene encoding threonine-insensitive bifunctional aspartate kinase/HMS dehydrogenase mutant (Walther et al., 2017a). The main glucose uptake pathway in *Escherichia coli* via the phosphoenolpyruvate-dependent glucose-specific phosphotransferase system (Liang et al., 2015) is marked by a bold arrow. Red crosses mark chromosomal gene deletion, and the red arrow indicates chromosomal overexpression. Bluish-green arrows mark the enzyme activities of synthetic homoserine-dependent pathways provided by plasmid-based expression.

Aided by synthetic biology and enzyme engineering, we and others have previously reported three artificial biosynthetic pathways for the aerobic, microbial production of DHB starting from the widely abundant and inexpensive sugar glucose (Walther et al., 2017b; Walther et al., 2015; Walther et al., 2017a). It is of note, however, that all the new routes are fully compatible with the use of other sugars (e.g., xylose, mannose, sucrose) or alcohols (e.g., methanol, ethylene glycol) as starting carbon sources, as all DHB pathways start from naturally occurring metabolites (homoserine, malate, or glyoxylate/acetyl-CoA). The different metabolic routes were tested in *Escherichia coli.* The highest reported titers (7.9 ± 0.01 mM) and yields (0.10 ± 0.01 mol_DHB_ mol_Glucose_^−1^) in shake-flask cultivations with glucose as carbon source have been achieved with the DHB pathway proceeding through the characteristic natural intermediate (L)-homoserine (Frazão et al., 2018). This route enables DHB synthesis via sequential deamination of (L)-homoserine by homoserine transaminase activity and reduction of 2-oxo-4-hydroxybutyrate (OHB) by an OHB reductase activity (Figure 1). In a previous study, we reported the construction of the highly active NADH-dependent OHB reductase Ec.Mdh^5Q^ (Frazão et al., 2018). The mutant variant descends from the parent NAD^+^-dependent (L)-malate dehydrogenase from *E. coli* (Ec.Mdh) and incorporates five mutations (I12V:R81A:M85Q:D86S:G179D) to yield the desired synthetic activity. However, the typical intracellular ratios of [NADH]/[NAD^+^] and [NADPH]/[NADP^+^] in *E. coli* cells cultivated under aerobic conditions are 0.03 and 60, respectively (Bennett et al., 2009), which suggests that the utilization of NADPH as a cofactor in aerobic reduction processes may be more favorable. Therefore, employing an enzyme bearing NADPH-dependent OHB reductase activity could provide a strong advantage in terms of pathway performance.

In the absence of annotated naturally occurring enzymes with NADPH-dependent OHB reductase activity, we chose NADH-dependent Ec.Mdh^5Q^ as the template enzyme to engineer the required activity. Previous studies have focused on engineering the nicotinamide cofactor specificity of oxidoreductases following rational approaches [as reviewed by Chánique and Parra (2018)] and typically rely on introducing amino acid substitutions in the co-enzyme binding site. Following this logic, we used comparative sequence and structural analyses supported by the use of a structure-guided web tool (Cahn et al., 2017) to engineer NADPH-dependent OHB reductase activity. The best-performing mutant, assessed by *in vitro* analysis, was subsequently introduced into a strain that produced DHB via the synthetic homoserine pathway. To demonstrate the full potential of our engineered NADPH-dependent OHB reductase variant and to prevent NADPH becoming a limiting factor in product synthesis, we transferred the DHB pathway into NADPH-overproducing *E. coli* strains (Figure 1) expressing the improved transaminase variant *Ec.alaC*_*A142P:Y275D*_ and showed a 50% increased DHB yield in shake-flask cultivations with glucose as the only carbon source for the most advanced strain.

## 2 Materials and methods

### 2.1 Reagents and chemicals

Unless stated otherwise, chemicals and solvents were purchased from Sigma-Aldrich (Darmstadt, Germany). Restriction enzymes and kits for plasmid DNA isolation, gel DNA extraction, and PCR clean-up were purchased from NEB (Frankfurt am Main, Germany) and used according to the manufacturer’s instructions. Primers were purchased from Sigma-Aldrich. Sanger sequencing was carried out by Genewiz (Leipzig, Germany).

### 2.2 Strains and plasmids

All strains and plasmids used in this study are listed in Tables 1, 2.

**TABLE 1:** Plasmids used in this study.

Plasmid	Relevant characteristic	Reference/Origin
pKD4	R6K γ ori, FRT-flanked Kan^R^, and Amp^R^	Datsenko and Wanner (2000)
pKD46	pSC101 ori, araC-P_araB_, λ-Red recombinase system (*γ, β, exo*, λ tL3 terminator), and Amp^R^	Datsenko and Wanner (2000)
pCP20	pSC101 ori, Amp^R^, Cm^R^, and Flp	Cherepanov and Wilfried (1995)
*In vitro* studies		
pET28a(+)	*f1* origin, Kan^R^, and T7 promoter	Novagen™
pET28-*Ec-Mdh* ^*5Q*^	pET28a(+) derivative N-terminal His-tagged *Ec.mdh*_*R81A:M85Q:I12V:G179D:D86S*_	Frazão et al. (2018)
pET28-*Ec-Mdh* ^*5Q*^ *-D34G*	pET28a(+) derivative with N-terminal His-tagged *Ec.mdh*_*R81A:M85Q:I12V:G179D:D86S:D34G*_	This study
pET28-*Ec-Mdh* ^*5Q*^ *-D34G:I35K*	pET28a(+) derivative with N-terminal His-tagged *Ec.mdh*_*R81A:M85Q:I12V:G179D:D86S:D34G:I35K*_	This study
pET28-*Ec-Mdh* ^*5Q*^ *-D34G:I35R*	pET28a(+) derivative with N-terminal His-tagged *Ec.mdh*_*R81A:M85Q:I12V:G179D:D86S:D34G:I35R*_	This study
pET28-*Ec-Mdh* ^*5Q*^ *-D34G:I35S*	pET28a(+) derivative with N-terminal His-tagged *Ec.mdh*_*R81A:M85Q:I12V:G179D:D86S:D34G:I35S*_	This study
pET28-*Ec-Mdh* ^*5Q*^ *-D34G:I35T*	pET28a(+) derivative with N-terminal His-tagged *Ec.mdh*_*R81A:M85Q:I12V:G179D:D86S:D34G:I35T*_	This study
*In vivo* DHB production		
pZA23	*p15A* origin, Kan^R^, and P_A1lacO-1_ promoter	Expressys
pZA23-HS1-5Q	pZA23 derivative carrying *Ec.thrA*_*S345F*_, *Ec.aspC*, *Ec.mdh* ^*5Q*^, and *Ec.ppc*_*K620S*_	Frazão et al. (2018)
pZA23-HS1-7Q	pZA23 derivative harboring *Ec.thrA*_*S345F*_, *Ec.aspC, Ec.mdh* ^*7Q*^, and *Ec.ppc*_*K620S*_	This study
pZA23-HS2-5Q	pZA23 derivative harboring *Ec.thrA*_*S345F*_, *Ec.alaC*_*A142P:Y275D*_, *Ec.Mdh* ^*5Q*^, and *Ec.ppc*_*K620S*_	Frazao (2019)
pZA23-HS2-7Q	pZA23 derivative harboring *Ec.thrA*_*S345F*_, *Ec.alaC*_*A142P:Y275D*_, *Ec.mdh* ^*7Q*^, and *Ec.ppc*_*K620S*_	This study

**TABLE 2:** *Escherichia coli* strains used in this study.

Strain	Genotype	Reference/Origin
NEB^®^ 5-alpha	*E. coli fhuA2 Δ(argF-lacZ)U169 phoA glnV44 Φ80Δ (lacZ)M15 gyrA96 recA1 relA1 endA1 thi-1* hsdR17	NEB™
BL21 (DE3)	*E. coli fhuA2 [lon] ompT gal (λ DE3) [dcm] ∆hsdS*	NEB™
MG1655	*F* ^*−*^ *λ* ^*-*^ *ilvG- rfb-50 rph-1*	ATCC
bWL1221	*E. coli* MG1655 *lldD* ^*proD-kan*^	INSA Toulouse
EcHS0	*E. coli* MG1655 *ΔthrB ΔmetA ΔldhA*	This study
EcHS1	EcHS0 with pZA23-HS1-5Q (*Ec.aspC*, *Ec.mdh* ^*5Q*^ *)*	This study
EcHS2	EcHS0 with pZA23-HS1-7Q (*Ec.aspC*, *Ec.mdh* ^*7Q*^ *)*	This study
EcHS3	EcHS0 with pZA23-HS2-5Q (*Ec.alaC*_*A142P:Y275D*_, *Ec.mdh* ^*5Q*^ *)*	This study
EcHS4	EcHS0 with pZA23-HS2-7Q (*Ec.alaC*_*A142P:Y275D*_, *Ec.mdh* ^*7Q*^ *)*	This study
EcHS5	EcHS3 with *ΔpfkA*	This study
EcHS6	EcHS3 with *ΔsthA*	This study
EcHS7	EcHS3 with *pntAB* ^*proD*^	This study
EcHS8	EcHS7 with *ΔsthA*	This study
EcHS9	EcHS4 with *ΔpfkA*	This study
EcHS10	EcHS4 with *ΔsthA*	This study
EcHS11	EcHS4 with *pntAB* ^*proD*^	This study
EcHS12	EcHS11 with *ΔsthA*	This study

### 2.3 Media

For cloning procedures, protein production, and cell recovery from glycerol stocks (30% v/v) kept at −80°C, cells were cultivated in lysogeny broth (LB) medium (10 g L^−1^ tryptone, 5 g L^−1^ yeast extract and 10 g L^−1^ NaCl). LB agar plates were prepared by adding 20 g L^−1^ agar-agar to liquid LB.

For DHB production studies, cells were cultivated in M9 mineral medium (Walther et al., 2017b), containing 20 g L^−1^ glucose, 100 mM MOPS (pH adjusted to 7 with KOH), 18 g L^−1^ Na_2_HPO_4_·12H_2_O, 3 g L^−1^ KH_2_PO_4_, 0.5 g L^−1^ NaCl, 2 g L^−1^ NH_4_Cl, 0.5 g L^−1^ MgSO_4_·7 H_2_O, 0.015 g L^−1^ CaCl_2_·2H_2_O, 0.010 g L^−1^ FeCl_3_, 0.012 g L^−1^ thiamine HCl, and trace elements (0.4 mg L^−1^ Na_2_EDTA·2H_2_O, 1.8 mg L^−1^ CoCl_2_·6H_2_O, 1.8 mg L^−1^ ZnCl_2_SO_4_·7H_2_O, 0.4 mg L^−1^ Na_2_MoO_4_·2H_2_O, 0.1 mg L^−1^ H_3_BO_3_, 1.2 mg L^−1^ MnSO_4_·H_2_O, and 1.2 mg L^−1^ CuCl_2_·2H_2_O). (L)-methionine and (L)-threonine were added at a final concentration of 0.2 g L^−1^ each to compensate for auxotrophies of the production strains. Where required, antibiotics were added at the following final concentrations: carbenicillin, 100 mg L^−1^, and kanamycin sulfate, 50 mg L^−1^.

### 2.4 Site-directed mutagenesis, gene expression, and protein purification for *in vitro* enzyme studies

Site-directed mutagenesis was performed via inverse PCR (Zheng et al., 2004) with primer pairs listed in Supplementary Table S1 using 6 ng of vector pET28-*Ec.mdh* ^*5Q*^ as a template (Frazão et al., 2018). The PCR reaction was performed with Q5 polymerase (NEB). Compared to the manufacturer’s instructions, the PCR cycle number was reduced to 19. After treatment with DpnI enzyme (NEB) to remove residual template DNA, plasmids were transformed into *E. coli* NEB^®^ 5-alpha, and respective mutations were verified by DNA Sanger sequencing. The constructed plasmids are listed in Table 1.

N-terminally 6x-His-tagged enzymes were produced in *E. coli* BL21 (DE3) cells harboring respective pET28a expression vectors. A volume of 50 mL of lysogeny broth (LB) medium supplemented with kanamycin in a 250 mL unbaffled shake flask was inoculated at an initial optical density at 600 nm (OD_600_) of 0.2 from an overnight LB culture. The culture was incubated at 37°C and 220 rpm. Heterologous protein expression was induced when OD_600_ = 0.6 by the addition of 1 mM isopropyl β-D-1-thiogalactopyranoside (IPTG) to the medium. When an OD_600_ of 2 was reached, cells were harvested by centrifugation (10 min, 4,000 ×g, 4°C), and the cell pellets were stored at −20°C until further analysis. To purify the His-tagged protein, frozen cell pellets were thawed on ice, resuspended in 50 mM HEPES buffer containing 300 mM NaCl (pH = 7.5), and then disrupted using a sonicator (UDS 751, TOPAS, Germany, 4 × 30 s, 30% amplitude). Cell debris was removed from the soluble protein fraction by centrifugation (17,500 ×g, 15 min, 4°C). Upon washing of the Talon™ Cobalt affinity resin (Clontech, United States) according to the supplier’s instructions, the crude extract was added to the resin and incubated in a tube rotator (VWR) at room temperature for 20 min. After binding, a washing step with 50 mM HEPES buffer containing 300 mM NaCl (pH = 7.5) and a subsequent washing step with the same buffer but additionally containing 15 mM imidazole (pH = 7.5) were carried out. Afterward, the protein bound to the resin was eluted with 200 mM imidazole in 50 mM HEPES buffer containing 300 mM NaCl (pH = 7.5) in a final volume of 500 µL. The concentration of purified protein was determined using the Bradford assay (Rotiquant^®^, Carl Roth), usually yielding a protein concentration of 3–4 mg mL^−1^ after purification.

### 2.5 OHB synthesis

OHB was prepared enzymatically starting from (L)-homoserine in a reaction catalyzed by the (L)-amino acid oxidase from *Crotalus adamanteus* (Sigma-Aldrich, A9253) as described by Wellner and Lichtenberg (1971). The reaction mix contained 125 mM (L)-homoserine, 100 mM Tris-HCl (pH = 7.8), and 4,374 U mL^−1^ catalase from *Aspergillus niger* (Sigma-Aldrich, C3515), and 4.7 U mL^−1^ (L)-amino acid oxidase. Control reactions were performed with the same reaction mix but in the absence of (L)-homoserine or (L)-amino acid oxidase, respectively. The reaction was performed for 4.5 h at 37°C and 220 rpm. After incubation on ice for 1 h, enzymes were removed using Amicon^®^ Ultra Centrifugal filters (cut-off, <10 kDa; Merck, Germany) for 45 min at 4°C. Quantification of OHB was performed based on a ketone calibration curve (0–300 mM pyruvate). A volume of 100 µL of standard/sample was mixed with 1 mL of a solution containing 1 M sodium arsenate and 1 M boric acid (pH = 6.5). After incubation at room temperature for 30 min, the absorbance at 325 nm was measured. The absence of homoserine was further confirmed by high-performance liquid chromatography (HPLC) analysis.

### 2.6 OHB reductase assay

Enzymatic assays with purified enzyme were conducted at 37°C in 96-well flat-bottomed microtiter plates with a final reaction volume of 250 µL per well. The reaction kinetics were monitored in a microplate reader (NanoQuant Plate™, Infinite M200 PRO, TECAN) by following the characteristic absorption of NAD(P)H at 340 nm. The reaction mixture contained 0.25 mM NAD(P)H, 60 mM Hepes (pH 7.0, adjusted with 5 M KOH), 5 mM MgCl_2_, 50 mM KCl, and appropriate amounts of the purified enzyme. Reactions were started by adding 2 mM OHB to assess the cofactor preference of the constructed enzyme variants. To determine the kinetic constants on the substrate of Ec.Mdh^5Q^ and top-performing NADPH-dependent OHB reductase, specific activities were determined at variable OHB concentrations (0.005–10 mM) and 0.25 mM of the preferred co-substrate. In order to estimate the kinetic constants on the cofactors, specific activities were determined at fixed amounts of substrate (OHB, 2 mM) and variable amounts of NAD(P)H (0.03–1 mM). Experimental data were fitted to the Michaelis–Menten model or to the substrate inhibition model using non-linear regression (Curve fitting tool, MATLAB R2021a). One unit (U) is defined as the amount of enzyme that catalyzes the conversion of 1 µmol of NAD(P)H per minute at pH 7.0 and 37°C. K_m,app_ [mM] is defined as the apparent Michaelis–Menten constant, K_i_ [mM] is the substrate inhibition constant, v_max,app_ [U mg^−1^] is the apparent maximum reaction speed, and k_cat,app_ is the apparent catalytic constant [s^−1^]. The catalytic efficiency is described by k_cat_/K_m_ [mM s^−1^] (Chmiel, 2018). The specificity for NADPH is described as (k_cat,app_/K_m,app_)_NADPH_/(k_cat,app_/K_m,app_)_NADH_, calculated from the mean values of k_cat_ and K_m_ (Cahn et al., 2017). We furthermore define the overall catalytic efficiency with respect to substrate conversion in the presence of the preferred cofactor as (k_cat,app_/K_m,app_)_OHB_ × (k_cat,app_/K_m,app_)_NAD(P)H_.

### 2.7 Plasmid construction for *in vivo* DHB biosynthesis

All plasmids constructed and used for *in vivo* DHB synthesis are listed in Table 1. The vectors are based on the pZA23 backbone of the pZ expression system (Expressys). Plasmids pZA23-HS1-5Q (*Ec.aspC, Ec.mdh* ^*5Q*^) and pZA23-HS2-5Q (*Ec.alaC*_*A142P:Y275D*_, *Ec.mdh* ^*5Q*^) were a kind gift of Prof. J. M. François from Toulouse Biotechnology Institute, INSA Toulouse, France, and served as the basis for gene replacements. To replace OHB reductase-encoding gene *Ec.mdh* ^*5Q*^, the gene *Ec.mdh* ^*7Q*^ was first amplified by PCR from the corresponding pET28 vector using the primers TW2949 and TW2427 listed in Supplementary Table S1, thereby introducing 5′ overhangs containing NotI and XbaI restriction sites. Backbone vectors pZA23-HS1-5Q and pZA23-HS2-5Q and insert were digested with the restriction enzymes NotI and XbaI (NEB). The digested backbone was further treated with Antarctic phosphatase (NEB). After DNA purification by gel extraction (Gel Extraction Kit, NEB), the backbone and insert were ligated using T4 DNA ligase (NEB) according to the provider’s protocol. After verification via sequencing, plasmids were transformed into appropriate host strains.

### 2.8 Strain construction

All *E. coli* strains constructed and used are listed in Table 2. Chromosomal gene deletions in *E. coli* MG1655 were introduced by P1vir phage transduction (Lennox, 1955) using single-gene knockout mutants from the Keio collection (Baba et al., 2006) as donor strains. After transduction, the kanamycin resistance cassettes were removed using flippase (FLP) recombinase-catalyzed excision. The FLP recombinase was expressed from pCP20 (Cherepanov and Wilfried, 1995). Gene deletions and successful removal of resistance cassettes were confirmed by diagnostic PCR (Primers listed in Supplementary Table S1) using DreamTaq polymerase (ThermoFisher Scientific), following the protocol provided by the manufacturer. Afterward, a new round of chromosomal modification was initiated, and the procedure was repeated until all target deletions were introduced.

For chromosomal overexpression of *Ec.pntAB*, the native chromosomal 5′-untranslated region of the gene was replaced by the insulated constitutive promoter proD (Davis et al., 2011) via PCR-mediated λ-Red recombination following the protocol of Datsenko and Wanner (2000). The FRT-kan-FRT cassette fused to proD promoter sequence with 50 bp 5′-extensions homologous to the target genomic locus was amplified by PCR (primers are listed in Supplementary Table S1) from the genomic DNA of strain bWL1221 in our lab collection (kindly provided by Prof. J. M. François from Toulouse Biotechnology Institute, INSA Toulouse, France). Recipient cells were transformed with the helper plasmid pKD46 (Datsenko and Wanner, 2000) for expression of λ-Red recombination genes and with linearized, gel-purified PCR product. Successful integration was confirmed by colony PCR, as described above. After kan-cassette removal using FLP recombinase expressed from pCP20, the chromosomal promoter exchange was furthermore confirmed via DNA sequencing.

### 2.9 Shake-flask cultivation for DHB production

All cell cultivations were performed at 37°C, 220 rpm in an orbital shaker (Ecotron, Infors). First, pre-cultures were inoculated with a single colony picked from an LB agar plate and cultivated in 3 mL LB media (15 mL Falcon tube lying flat), and 50 μg mL^−1^ kanamycin was added to the strains harboring pZA23 plasmids. After 8 h, a volume of 0.5 mL of the first pre-culture was transferred to 10 mL of M9 mineral medium, supplemented with (L)-methionine (0.2 g L^−1^), (L)-threonine (0.2 g L^−1^), and kanamycin. After 16 h of cultivation, cells were harvested by centrifugation in a table-top centrifuge (5 min, 6,000 ×g, room temperature). The main cultures were carried out in 25 mL M9 media supplemented with (L)-methionine (0.2 g L^−1^), (L)-threonine (0.2 g L^−1^), and kanamycin in 250 mL baffled shake flasks. The main cultures were inoculated with the harvested cells at a starting OD_600_ of 0.2. When an OD_600_ of ∼0.6 was reached, 1 mM IPTG was added to induce the expression of pathway genes. Samples were regularly withdrawn and centrifuged (2 min, 16,000 ×g, room temperature), and the supernatant was kept at −20°C until further analysis.

### 2.10 Analytical methods

Extracellular metabolites (glucose, DHB, acetate, and lactate) in supernatant samples from cultivations were analyzed using HPLC. The samples (1 mL) were filter sterilized with 0.2 µm filters, transferred into 2 mL HPLC sample vials, and subsequently analyzed with the Dionex UltiMate 3000 UHPLC system (Thermo Scientific). The device was equipped with an RI and UV/Vis detector. For separation, a Rezex™ ROA-Organic Acid H^+^ (8%) column (Phenomenex) with a size of 300 mm × 7.8 mm was used, protected by a SecurityGuard™ Carbo H^+^ pre-column (4 mm × 3 mm, Phenomenex). A sample volume of 20 µL was injected, and analytes were eluted using 0.5 mM H_2_SO_4_ as mobile phase, with a flow rate of 0.5 mL min^−1^. The column oven temperature was set to 80°C, and the temperature of the autosampler was set to 6°C.

Depending on the concentration range, the presence of DHB was verified by LC/MS analyses using our previously described method (Frazão et al., 2023). The LC/MS platform consists of a Vanquish and a Thermo Scientific™ Q Exactive™ Focus (ThermoFisher Scientific), controlled by Xcalibur software (version 2.1, ThermoFisher Scientific). Separation by liquid chromatography was achieved using a Rezex RoA-organic acid H^+^ (8%) resin-based column preceded by a SecurityGuard guard cartridge (Phenomenex) held at 80°C with 0.1% formic acid as the mobile phase. The temperature of the autosampler was kept at 6°C, the injection volume was 20 µL, and an isocratic flow of 0.4 mL min^−1^ was adjusted. Peak areas were corrected for the contribution of all naturally abundant isotopes using the software IsoCor (version 2.2.0) (Millard et al., 2012).

### 2.11 Computational methods

Multiple sequence alignment of NAD(P)H-dependent malate dehydrogenases and lactate dehydrogenases was performed using MAFFT (v 7.525) provided by EMBL-EBI (Madeira et al., 2024). UniProt identifiers of all proteins used in the alignment are listed in the supplementary material. Structure alignments were performed in PyMol v2.5.1 (http://www.pymol.org/pymol). Three-dimensional structures were retrieved from the Protein Data Bank (PDB) or predicted with AlphaFold Colab (Jumper et al., 2021).

## 3 Results

### 3.1 Strategy for the design of NADPH-dependent OHB reductase activity

**FIGURE 2:**
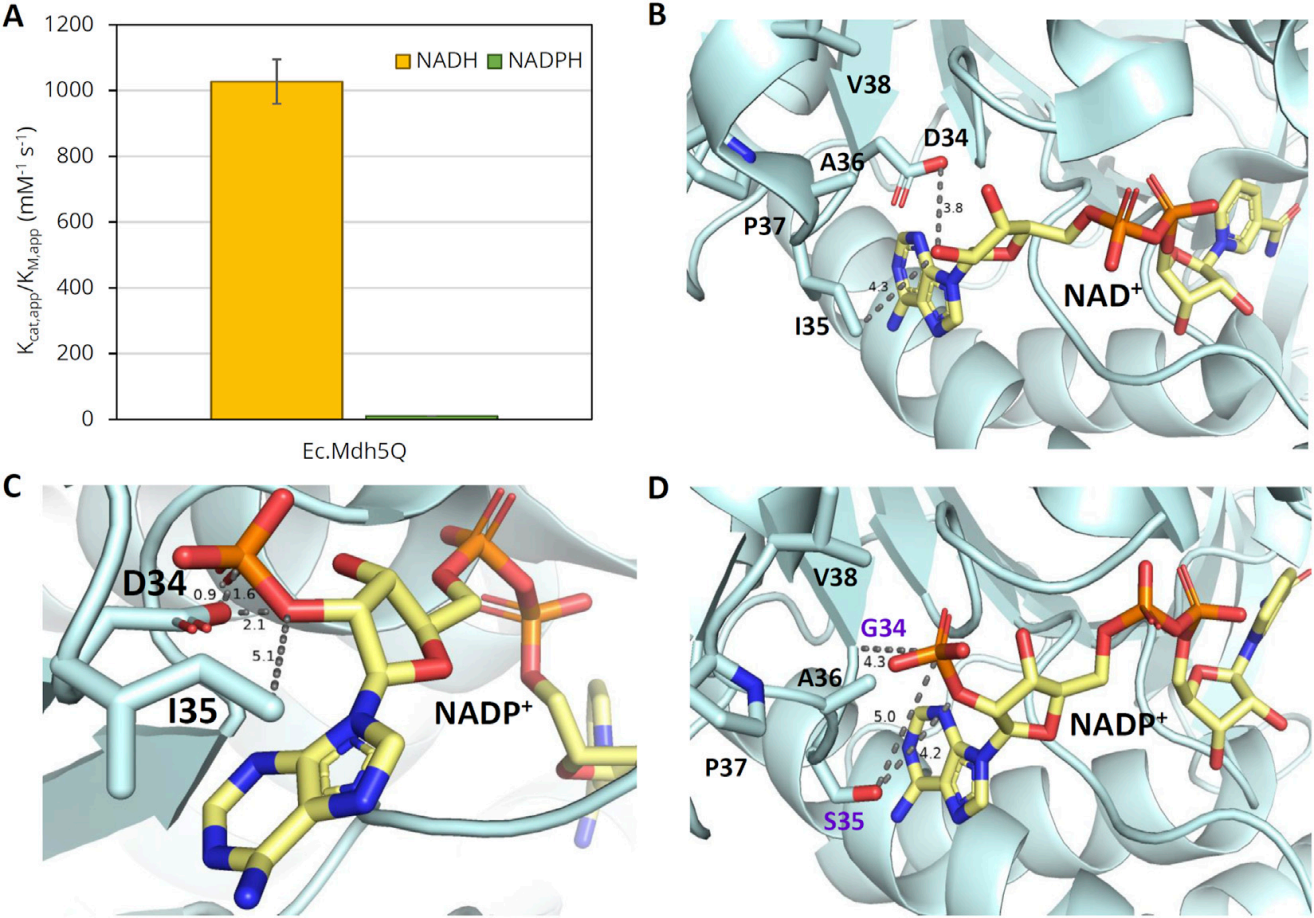
Specific activity of Ec.Mdh^5Q^ on OHB with NAD(P)H and 3D-protein structures with NAD^+^ or NADP^+^. **(A)** Catalytic efficiency (k_cat,app_/K_M,app_) of Ec.Mdh^5Q^ with NAD(P)H. Enzymatic activity was measured with purified enzyme at 37°C and pH 7.0 in 96-well flat-bottomed microtiter plates with 2 mM OHB and 0.25 mM NAD(P)H. The reactions were followed by monitoring the NAD(P)H absorption at 340 nm. Error bars indicate the standard deviation of the mean (n = 2). **(B)** X-ray structure of Ec.Mdh with bound NAD^+^ (Pdb code: 1emd). Side chains of amino acids in the selectivity control loop are shown (positions 34–38). **(C)** X-ray structure of Ec.Mdh with superimposed NADP^+^. The figure was generated by the structural alignment of Ec.Mdh with bound NAD (Pdb code: 1emd) and the malate dehydrogenase from *Flaveria bidentis* (Fb.Mdh) with bound NADP (Pdb code: 1civ). The structures of Fb-Mdh and NAD were omitted from the representation. **(D)** Model structure of Ec.Mdh D34G:I35S with superimposed NADP^+^. The figure was generated by structural alignment of the mutant enzyme model [generated with AlphaFold Colab (Jumper et al., 2021); mutations relative to wt enzyme are marked in violet] and the crystal structure of Fb.Mdh with bound NADP. Subfigures **(B–D)** were generated in PyMol v2.5.1 (http://www.pymol.org/pymol). The distance between selected residues is shown in Å. The carbon ribbon of cofactors NAD(P)^+^ is shown in pale yellow. The color scheme of NAD(P)^+^ and relevant amino acid side chains (position 34–38) is oxygen – red, nitrogen – blue, and sulfur – orange.

We have previously engineered a highly active OHB reductase using the NAD^+^-dependent (L)-malate dehydrogenase from *E. coli* (Ec.Mdh; UniProtKB code P61889) as a template enzyme. The best-performing variant, Ec.Mdh^5Q^, contains five point mutations (I12V:R81A:M85Q:D86S:G179D) and displays a 108-fold higher catalytic efficiency with NADH than with NADPH (Figure 2A). Because the typical intracellular ratios of [NAD(P)H]/[NAD(P)] in *E. coli* (Bennett et al., 2009) indicate that NADPH should be preferentially used as a cofactor in reduction processes under aerobic conditions, we set out to engineer an NADPH-dependent OHB reductase using Ec.Mdh^5Q^ as the template enzyme.

NAD^+^-dependent (L)-malate dehydrogenases (cytosolic; Mdh type 1 family) belong to the large superfamily of (L)-Mdh/(L)-Ldh enzymes, which further includes NADP^+^-dependent enzymes of identical function (chloroplastic; Mdh type 2 family) and NAD^+^-dependent (L)-lactate dehydrogenases (Ldh). Multiple sequence alignments between several members of the superfamily of Mdh and Ldh enzymes revealed a strong conservation of the primary protein structure (Supplementary Figure S1). Previous studies elucidated the crucial role of a loop region in a conserved cofactor binding motif of the Rossman fold for cofactor discrimination (Holmberg et al., 1999; Nishiyama et al., 1993; Feeney et al., 1990; Rossmann et al., 1974). Multiple sequence alignments showed that this region corresponds to the amino acid residues at positions 34 and 35 in Ec.Mdh (Supplementary Figure S1). Of crucial interest in Ec.Mdh is the active site residue Asp34, which is pivotal for conferring cofactor specificity because its negative charge has previously been shown to result in electrostatic repulsion of the 2′-phosphate group of NADP(H) (Nishiyama et al., 1993; Feeney et al., 1990). Visual inspection of the X-ray crystal structure of Ec.Mdh (PDB code with bound NAD^+^: 1emd, Figure 2B) bound with superimposed NADPH (Figure 2C) further revealed a potential steric clash between the aspartate residue at position 34 and the 2′-phosphate moiety of NADPH. Multiple sequence alignment analyses showed strict conservation at the corresponding position in NADP^+^-dependent malate dehydrogenases with a glycine residue (Supplementary Figure S1). Replacement of Asp34 residue in Ec.Mdh by the smaller and uncharged residue glycine, therefore, seemed crucial to accommodate the larger NADP(H) and to remove the unfavorable electrostatic interaction with NADP(H). In addition, residue Ile35 is of interest for switching the cofactor preference of Ec.Mdh. Due to its close proximity to the 2′-phosphate group of NADPH (Hall and Banaszak, 1993), the amino acid residue located at position 35 is prone to form an electrostatic interaction or a hydrogen bond with the cofactor. Multiple sequence alignment of natural NADPH-dependent Mdh enzymes reveals complete conservation of a potentially hydrogen-bond donating serine residue at the position corresponding to Ile35 in Ec.Mdh. Therefore, we hypothesized that the isoleucine residue at position 35 should be exchanged for serine. This idea was complemented by the structure-guided web tool CSR-salad (Cahn et al., 2017), which suggested an exchange of Ile35 by positively charged residues (Lys, Arg) or other polar uncharged amino acids (Thr) to allow for an electrostatic or polar interaction with the 2′-phosphate group of NADPH. Visual inspection of the predicted 3D structure of a respective Ec.Mdh D34G:I35S double mutant bound with NADP^+^ confirms the predicted absence of steric clashes in the selectivity control loop (Figure 2D).

### 3.2 *In vitro* analysis of OHB reductase mutants

**FIGURE 3:**
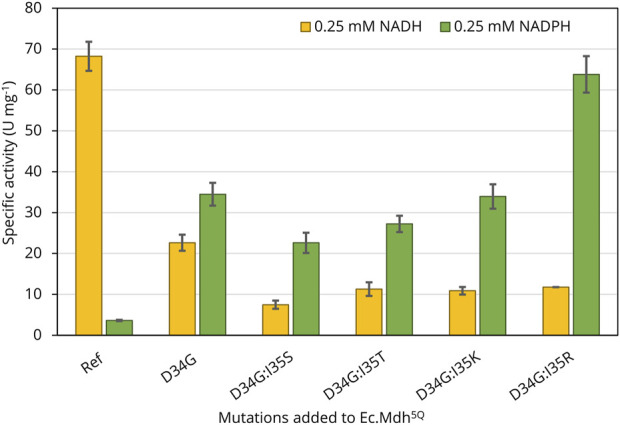
Specific NAD(P)H-dependent OHB reductase activity of engineered variants derived from Ec.Mdh^5Q^. The enzymes were produced from *E. coli* BL21 (DE3) harboring the respective pET28a expression vectors cultivated in 50 mL LB. Heterologous expression was induced at OD_600_ of 0.6 with 1 mM IPTG. Protein expression was carried out until OD_600_ = 2 was reached. Enzymatic assays were performed with purified enzyme at 37°C and pH 7.0 in 96-well flat-bottomed microtiter plates with 2 mM OHB and 0.25 mM NAD(P)H. The reactions were followed by monitoring the NAD(P)H absorption at 340 nm. Error bars indicate the standard deviation of the mean (n = 2).

Point mutations to switch cofactor preference were stepwise introduced into the *Ec.mdh* ^*5Q*^ gene by site-directed mutagenesis. The constructed N-terminally 6x-His-tagged variants were expressed from pET28a vectors transformed in *E. coli* BL21(DE3) cells. After purification, the specific activity of the purified variants was first quantified in the presence of OHB (2 mM) and either NADH or NADPH (0.25 mM) as cofactor. As shown in Figure 3, the template enzyme Ec.Mdh^5Q^ was highly active on OHB with NADH as a co-substrate (68 ± 4 U mg^−1^) but displayed low activity in the presence of NADPH (4 ± 0.2 U mg^−1^). For all of the engineered mutants, the cofactor preference (here defined as v_(NADPH)_/v_(NADH)_) was found to be altered. Replacement of the Asp34 residue by glycine resulted in comparable OHB reductase activity in the presence of both cofactors (v_(NADH)_ = 23 ± 2 U mg^−1^, v_(NADPH)_ = 35 ± 3 U mg^−1^), possibly indicating dual cofactor preference. The additional substitution of isoleucine at position 35 by serine, threonine, lysine, or arginine resulted in at least 2.5-fold higher activities in the presence of NADPH than NADH. With 64 ± 4.5 U mg^−1^, the specific OHB reductase activity of Ec.Mdh^5Q^ D34G:I35R (hereafter abbreviated as Ec.Mdh^7Q^) with NADPH as a cofactor was nearly six-fold higher than with NADH (12 ± 0.02 U mg^−1^). The mutant variant showed a similar specific activity on OHB when compared to that of Ec.Mdh^5Q^. Thus, Ec.Mdh^7Q^ was identified as the most promising NADPH-dependent OHB reductase enzyme, and its kinetic parameters on both cofactors and OHB were determined and compared to those of Ec.Mdh^5Q^.

The kinetic parameters of both enzyme variants are summarized in Table 3. The engineered variant Ec.Mdh^7Q^ exhibited more than three orders of magnitude higher specificity (defined as (k_cat_/K_m(NADPH)_)/(k_cat_/K_m(NADH)_) for NADPH than Ec.Mdh^5Q^ (16 and 0.01, respectively). No loss in OHB affinity or impairment of the overall catalytic efficiency ((k_cat_/K_m_)_OHB_ × (k_cat_/K_m_)_NAD(P)H_) was observed. We observed uncompetitive substrate inhibition of Ec.Mdh^7Q^ by OHB (K_i_ = 5.5 ± 0.5 mM). Substrate inhibition was found to be stronger than for Ec.Mdh^5Q^ (K_i_ = 31.9 mM ± 5.6 mM) (Frazão et al., 2018).

**TABLE 3:** Kinetic analysis of OHB reductases Ec.Mdh^5Q^ and Ec.Mdh^7Q^.

Enzyme	Ec.Mdh^5Q^	Ec.Mdh^7Q^
NADH^a^		
V_max,app_ (U mg^−1^)	67.68 (±3.12)	20.02 (±1.99)
k_cat,app_ (s^−1^)	36.48 (±1.68)	10.79 (±1.07)
K_m,app_ (mM)	0.04 (±0.004)	0.27 (±0.04)
k_cat,app_/K_m,app_ (mM^−1^ s^−1^)	1,027.40 (±67.46)	40.35 (±1.71)
NADPH^a^		
V_max,app_ (U mg^−1^)	5.56 (±0.64)	118.80 (±18.5)
k_cat,app_ (s^−1^)	2.99 (±0.35)	64.03 (±9.97)
K_m,app_ (mM)	0.32 (±0.04)	0.10 (±0.03)
k_cat,app_/K_m,app_ (mM^−1^ s^−1^)	9.50 (±0.05)	645.47 (±76.25)
OHB^b^		
V_max,app_ (U mg^−1^)	138.50 (±10.45)	154.80 (±6.30)
k_cat,app_ (s^−1^)	74.89 (±5.63)	83.43 (±3.40)
K_m,app_ (mM)	1.84 (±0.50)	1.08 (±0.19)
K_i_ (mM)	31.9 (±5.6)^c^	5.45 (±0.48)
k_cat,app_/K_m,app_ (mM^−1^ s^−1^)	44.91 (±15.18)	80.29 (±17.27)
Specificity		
(k_cat,app_/K_m,app_)_NADPH_/(k_cat,app_/K_m,app_)_NADH_	0.01	16.00
Overall catalytic efficiency		
(k_cat,app_/K_m,app_)_OHB_ × (k_cat,app_/K_m,app_)_NAD(P)H_	46,136	51,823

^a^ Specific OHB reductase activities were determined at fixed concentrations of substrate (OHB, 2 mM) and variable concentrations of NAD(P)H (1–0.03 mM).

^b^ Specific OHB reductase activities were determined at fixed concentrations (0.25 mM) of the preferred co-substrate NAD(P)H and variable amounts of OHB (10–0.005 mM).

^c^ Frazão et al. (2018).

Apparent kinetic constants (K_m,app_, V_max,app_) were estimated by fitting the experimental data to the Michaelis–Menten model using non-linear regression unless enzymes displayed substrate inhibition kinetics (Curve fitting tool, MATLAB R2021a). To calculate the apparent catalytic constant k_cat_,_app_, the molecular weight of one subunit of Ec.Mdh (32.337 kDa) was considered. Specificity and efficiency were calculated based on mean k_cat,app_ and K_m,app_ values. Enzyme assays were performed in biological duplicates. Ec.Mdh^5Q^ contains mutations I12V:R81A:M85Q:D86S:G179D.

Ec.Mdh^7Q^ additionally contains mutations D34G:I35R.

### 3.3 DHB production using NADPH-dependent OHB reductase

Next, the *in vivo* performance of the NADPH-dependent OHB reductase Ec.Mdh^7Q^ for DHB biosynthesis was investigated. To achieve DHB production from glucose, we selected *E. coli* MG1655 *ΔthrB ΔmetA ΔldhA* as a production host to ensure a sufficient supply of the homoserine precursor. The host was then equipped with a DHB pathway consisting of homoserine transaminase activity (catalyzed by Ec.AspC from *E. coli*), and NAD(P)H-dependent OHB reductase activity (either Ec.Mdh^5Q^ or Ec.Mdh^7Q^) expressed from a medium-copy plasmid pZA23 under the control of the ITPG-inducible P_A1lacO-1_ promoter. Constructed plasmids additionally carried threonine-insensitive bifunctional aspartate kinase/homoserine dehydrogenase (Ec.ThrA_S345F_) and the aspartate/malate insensitive phosphoenolpyruvate carboxylase variant Ec.Ppc_K620S_ (Frazão et al., 2018) (see the metabolic setting in Figure 1).

**FIGURE 4:**
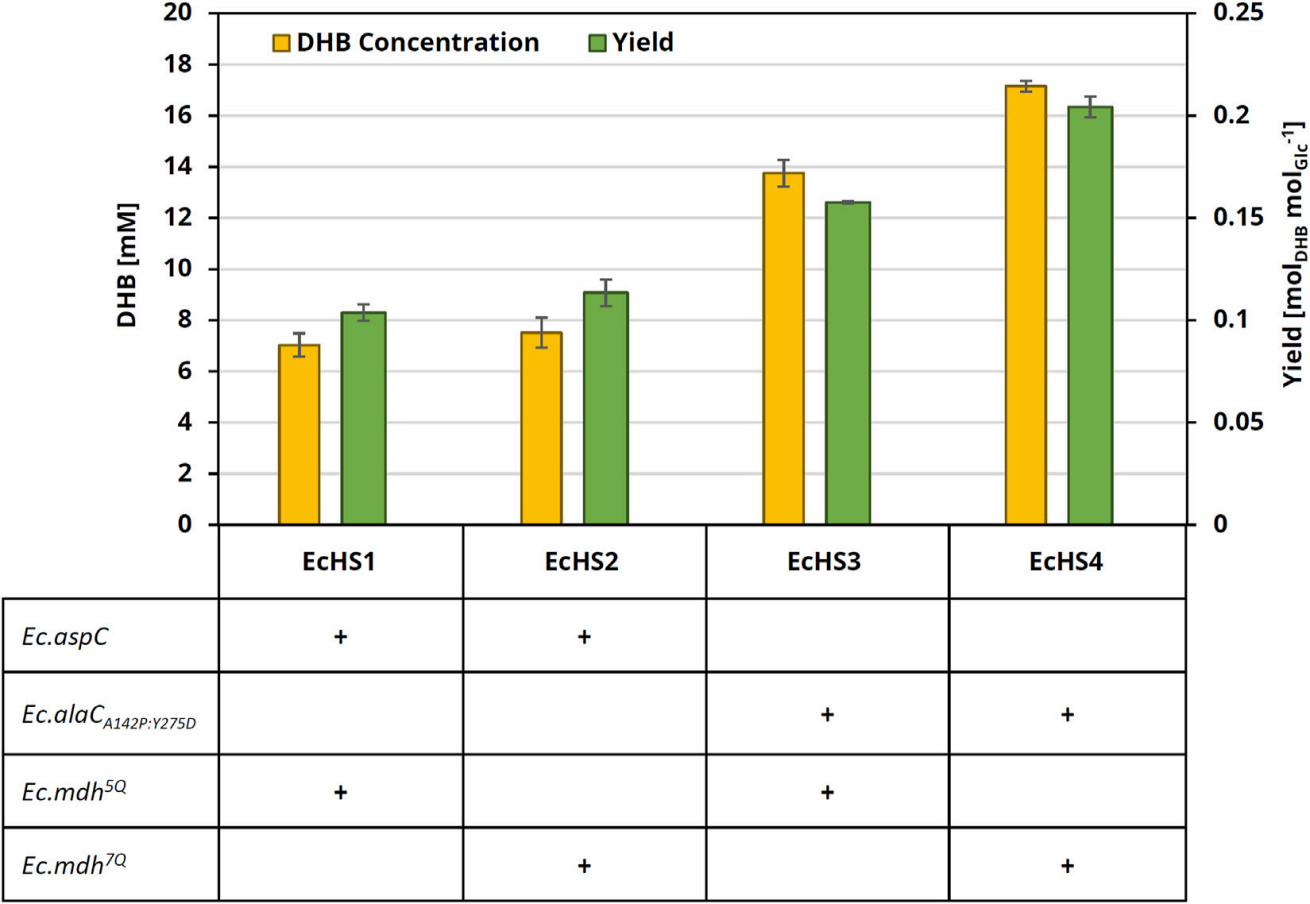
DHB concentration and yield after 24 h cultivation of engineered production strains. All strains are derived from the parental strain *E. coli* MG1655 *ΔthrB ΔmetA ΔldhA*, which was transformed with medium-copy DHB production plasmids expressing homoserine transaminase (*Ec.aspC or Ec.alaC*_*A142P:Y275D*_) and OHB reductase (*Ec.Mdh*_*5Q*_ or *Ec.Mdh*_*7Q*_). All plasmids further carry *Ec.thrA*_*S345F*_ and *Ec.ppc*_*K620S*_. Cultivation was performed at 37°C and 220 rpm in 250 mL baffled flasks containing 25 mL M9 medium with 20 g L^−1^ glucose, supplemented with 0.2 g L^−1^ (L)-methionine and 0.2 g L^−1^ (L)-threonine. The expression of pathway genes was induced at an OD_600_ of 0.6 with 1 mM IPTG. DHB titers and yields after 24 h of cultivation are shown. The experiments were performed in biological duplicates. Error bars indicate the standard deviation of the mean.

Producer strains were cultivated in 25 mL M9 mineral medium containing 20 g L^−1^ glucose. (L)-methionine and (L)-threonine were added to the medium (at a final concentration of 0.2 g L^−1^ each) to compensate for the host strain’s auxotrophies. Expression of pathway genes was induced by IPTG (1 mM) at the mid-exponential phase, and DHB production was quantified after 24 h of cell cultivation. Upon expression of Ec.Mdh^5Q^, we found the culture supernatant to contain 7.0 ± 0.5 mM DHB (EcHS1, Figure 4), reaching a product yield of 0.10 ± 0.004 mol_DHB_ mol_Glucose_^−1^ and a volumetric productivity of 0.29 ± 0.02 mmol_DHB_ L^−1^ h^−1^. Strain EcHS2 expressing Ec.Mdh^7Q^ was able to produce 7.5 ± 0.6 mM DHB. Because only a modest increase in DHB production was observed with NADPH-dependent OHB reductase, we speculated at this stage that homoserine transaminase activity was limiting. Indeed, purified Ec.AspC enzyme has previously been shown to display only low *in vitro* activity on (L)-homoserine (0.082 U mg^−1^), and it could not be saturated at substrate concentrations of up to 50 mM (L)-homoserine (Walther et al., 2017a; Walther et al., 2017b). Crucially, however, Bouzon and co-workers (2017) previously disclosed a homoserine transaminase (Ec.AlaC_A142P:Y275D_) with a much higher affinity for homoserine (K_m_ = 1.7 mM). Therefore, we reanalyzed the effect of the different OHB reductases when co-expressing Ec.AlaC_A142P:Y275D_ in the producer strains. Indeed, co-expression of Ec.Mdh^5Q^ and Ec.AlaC_A142P:Y275D_ (EcHS3) led to a DHB titer of 13.8 ± 0.5 mM, which constitutes roughly a 2-fold improvement over the use of Ec.AspC. This result confirms the homoserine transaminase activity of Ec.AspC as a rate-limiting step in the pathway, and we retained the transaminase Ec.AlaC_A142P:Y275D_ for all subsequent experiments. Replacement of Ec.Mdh^5Q^ by NADPH-dependent OHB reductase led to the production of 17.2 ± 0.2 mM (EcHS4), which represents a 25% improvement compared to the corresponding strain with the NADH-dependent variant. By co-expressing *Ec.alaC*_*A142P:Y275D*_ and *Ec.mdh* ^*7Q*^ genes, a maximum DHB product yield of 0.20 ± 0.005 mol_DHB_ mol_Glucose_^−1^ and a volumetric productivity of 0.72 ± 0.01 mmol_DHB_ L^−1^ h^−1^ were achieved. Analysis of the enantiomeric purity of DHB revealed the presence of only the L-form of the organic acid (data not shown).

### 3.4 Construction of NADPH over-producing strain for improved DHB production

To demonstrate the full potential of NADPH-dependent OHB reductase toward DHB production, we next engineered the host strain *E. coli* MG1655 *ΔthrB ΔmetA ΔldhA* toward increased NADPH supply. We selected multiple chromosomal targets previously shown to increase the intracellular availability of NADPH, including the deletion of the *Ec.pfkA* gene (encoding 6-phosphofructokinase I) to enhance flux through the pentose phosphate pathway (Chin and Cirino, 2011). We further altered the expression of the transhydrogenase system toward the formation of NADPH by deleting soluble pyridine nucleotide transhydrogenase Ec.SthA (NAD^+^ + NADPH → NADH + NADP^+^; Boonstra et al., 1999; Sauer et al., 2004) and/or overexpressing membrane-bound pyridine nucleotide transhydrogenase Ec.PntAB (NADH + NADP^+^ → NAD^+^ + NADPH; Clarke et al., 1986; Sauer et al., 2004). The resulting host strains were then used to characterize the impact of increased NADPH availability on DHB production with the NADPH-dependent OHB reductase. To investigate potential effects on DHB production that may be caused by altered homoserine availability, we additionally tested Ec.Mdh^5Q^.

**FIGURE 5:**
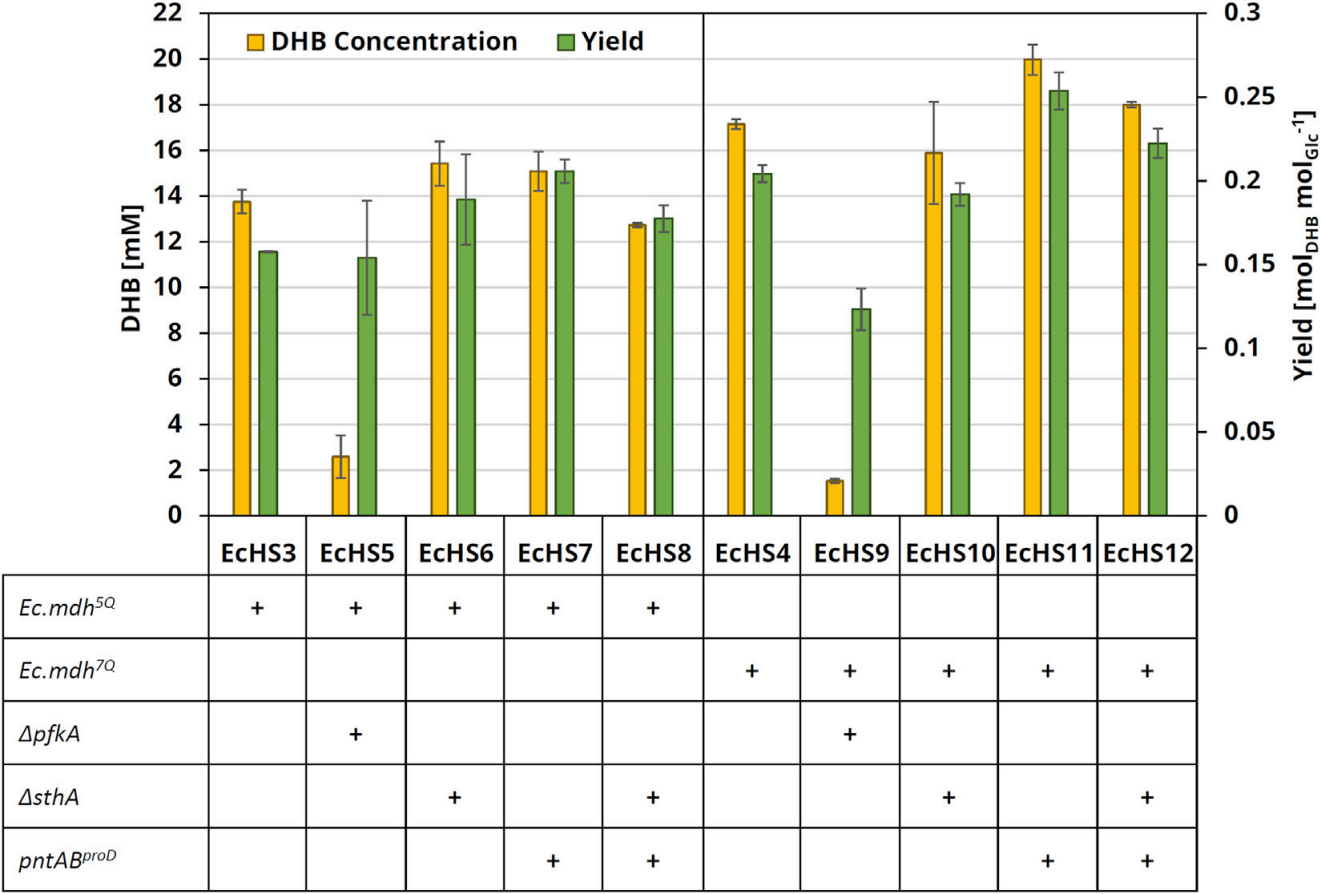
DHB concentration and yield after 24 h cultivation of engineered production strains with increased NADPH availability. All strains were derived from the parental strain *E. coli* MG1655 *ΔthrB ΔmetA ΔldhA*, which was transformed with medium-copy DHB production plasmids expressing homoserine transaminase (*Ec.alaC*_*A142P:Y275D*_) and OHB reductase (*Ec.mdh* ^*5Q*^ or *Ec.mdh* ^*7Q*^). All plasmids further carried *Ec.thrA*_*S345F*_ and *Ec.ppc*_*K620S*_ *.* Cultivation was performed at 37°C and 220 rpm in 250-mL baffled flasks containing 25 mL M9 medium with 20 g L^−1^ glucose, supplemented with 0.2 g L^−1^ (L)-methionine and 0.2 g L^−1^ (L)-threonine. Expression of pathway genes was induced at an OD_600_ of 0.6 with 1 mM IPTG. DHB titers and yields after 24 h of cultivation are shown. The experiments were performed in biological duplicates. Error bars indicate the standard deviation of the mean.

After 24 h of cultivation in glucose-containing mineral medium, the producer strains with the chromosomal deletion of the *Ec.pfkA* gene and expressing the NADH-dependent *Ec.mdh* ^*5Q*^ (EcHS5) exhibited a severe drop of both DHB concentration and yield when compared to the reference strain EcHS3 (Figure 5). This may be related to the observed growth defect caused by *Ec.pfkA* deletion (data not shown). With EcHS6 deleted for *Ec.sthA*, the DHB yield was equal to 0.19 ± 0.03 mol_DHB_ mol_Glucose_^−1^, which corresponds to a 19% increase when compared to reference strain EcHS3 with a yield of 0.16 mol ± 0.001 mol_DHB_ mol_Glucose_^−1^. Upon chromosomal overexpression of *Ec.pntAB* (EcHS7), the product yield was further increased to 0.21 ± 0.007 mol_DHB_ mol_Glucose_^−1^, which corresponds to a total increase of 30% compared to the reference strain (EcHS3). The additional deletion of *Ec.sthA* did not provide an advantage and indeed caused the DHB yield to drop to 0.18 ± 0.008 mol_DHB_ mol_Glucose_^−1^.

The production strains expressing *Ec.mdh* ^*7Q*^ displayed similar tendencies as the strains expressing *Ec.mdh* ^*5Q*^. However, the positive effect on DHB production upon chromosomal overexpression of *Ec.pntAB* was enhanced, yielding 0.25 ± 0.01 mol_DHB_ mol_Glucose_^−1^ after 24 h cultivation of EcHS11. Thus, increased NADPH availability via chromosomal overexpression of *pntAB* promoted DHB synthesis, particularly in the presence of the engineered NADPH-dependent enzyme variant.

To sum up, by co-expression of the improved homoserine transaminase (*Ec.alaC*_*A142P:Y275D*_) with the NADPH-dependent OHB reductase *Ec.mdh* ^*7Q*^ and by further modification of the production strain (*Ec.pntAB* ^*proD*^ *)*, it was possible to increase the DHB yield by 50% to 0.25 ± 0.01 mol_DHB_ mol_Glucose_^−1^ and reach a volumetric productivity of 0.83 ± 0.03 mmol_DHB_ L^−1^ h^−1^ within 24 h of batch cultivation of EcHS11.

### 3.5 Discussion

In previous work, we used a rational engineering approach to construct a highly active OHB reductase enzyme, catalyzing the last reaction step of the artificial homoserine-dependent DHB synthesis route (Frazão et al., 2018). Because we used the NAD^+^-dependent *E. coli* malate dehydrogenase (Ec.Mdh; UniProtKB code P61889) as a template to construct Ec.Mdh^5Q^ (I12V:R81A:M85Q:D86S:G179D), the reduction of OHB in the synthetic pathway relied on NADH as a cofactor. However, typical intracellular ratios of [NAD(P)H]/[NAD(P)] in *E. coli* (Bennett et al., 2009) indicate that the use of a NADPH-dependent OHB reductase provides a thermodynamic advantage. In this study, we aimed to replace the currently used NADH-dependent OHB reductase by an enzyme with NADPH-dependent activity.

Oxidoreductases with nicotinamide cofactor dependency exhibit a strong preference for either NAD(H) or NADP(H) (Chánique and Parra, 2018). In the large and complex (L)-malate/(L)-lactate dehydrogenase superfamily, most enzyme candidates of microbial origin rely on the NAD(H) cofactor system, typically showing only low residual activities with NADP(H) (Takahashi-Íñiguez et al., 2016; Brochier-Armanet and Madern, 2021). Whereas naturally existing NADP(H)-dependent Ldhs have not yet been identified (Richter et al., 2011), NADP(H)-preferring Mdhs exist in chloroplasts (Lemaire et al., 1996). Indeed, we initially envisaged the construction of a NADPH-dependent OHB reductase using the NADP^+^-dependent chloroplast malate dehydrogenase originating from *Sorghum bicolor* (Sb.chMdhP) as a template enzyme (Crétin et al., 1990). Because we did not succeed in the production of a soluble, active enzyme variant in *E. coli*, we altered our strategy and instead focused on switching the cofactor specificity of previously reported NADH-dependent OHB reductase variant Ec.Mdh^5Q^ toward NADPH.

Previous studies investigating the cofactor specificity of enzymes belonging to the (L)-malate/(L)-lactate dehydrogenase superfamily revealed that only a few amino acid residues are responsible for conferring the preference for either NAD(H) or NADP(H). In attempts to reverse the enzyme cofactor dependency, identifying crucial cofactor-discriminating key positions is usually followed by site-directed mutagenesis of the respective amino acid residues or a loop exchange approach (Chánique and Parra, 2018). Our present study confirms the crucial importance of the amino acid residues at positions 34 and 35 in Ec.Mdh in conferring cofactor preference. Upon replacing Asp34 and Ile35 with glycine and arginine, respectively, the cofactor specificity of the mutant Ec.Mdh^7Q^ was successfully altered from NADH to NADPH. Switching the cofactor specificity while maintaining comparable catalytic efficiency to that of the wild-type enzyme represents a significant challenge. In the majority of previous attempts of switching the cofactor specificity of oxidoreductases from NAD(H) to NADP(H), the catalytic efficiency of the mutant with NADP(H) was severely reduced, reaching less than 50% of the wild type’s catalytic efficiency with NAD(H) (Chánique and Parra, 2018). Although the catalytic efficiency of Ec.Mdh^7Q^ with NADPH was also reduced in the context of this study, it still reached more than 60% of that of the template enzymes with NADH. Further engineering efforts should be made to fully recover the activity of Ec.Mdh^7Q^ with NADPH compared to the template enzyme Ec.Mdh^5Q^ with NADH. More relevant for *in vivo* DHB pathway operation using the NADPH-dependent variant, the overall catalytic efficiency for OHB reduction in the presence of the preferred cofactor defined as (k_cat,app_/K_m,app_)_(OHB)_ × (k_cat,app_/K_m,app_)_(NAD(P)H)_ of the mutant Ec.Mdh^7Q^ (on OHB with NADPH) was comparable to the template enzyme Ec.Mdh^5Q^ (on OHB with NADH). Thus, the engineered Ec.Mdh^7Q^ was a promising candidate for *in vivo* application.

When we first replaced the NADH-dependent OHB reductase with our engineered NADPH-dependent variant for *in vivo* DHB production via the homoserine-dependent pathway, a positive effect could not be observed. Thus, we speculated that our current transaminase, Ec.AspC, was limiting the pathway flux (Figure 4). By exchanging Ec.AspC with the alanine aminotransferase double mutant Ec.AlaC_A142P:Y275D_ (Bouzon et al., 2017) and co-expression with the NADH-dependent Ec.Mdh^5Q^, we could indeed show a two-fold improvement in DHB production, reaching a concentration of 13.8 ± 0.5 mM. Upon co-expression of Ec.AlaC_A142P:Y275D_ and Ec.Mdh^7Q^, we could even reach up to 17.2 ± 0.2 mM DHB and a product yield of 0.20 ± 0.005 mol_DHB_ mol_Glucose_^−1^ after 24 h of cultivation. The results show the superior *in vivo* performance of the engineered NADPH-dependent OHB reductase variant but also clearly indicate the crucial role of sufficient transaminase activity for efficient pathway operation. However, it must be considered that the affinity of the engineered OHB reductase variant for the synthetic substrate might still be insufficient (K_m_ = 1.08 ± 0.19 mM), as intracellular OHB concentrations are expected to be in the range of sub-mM levels (Walther et al., 2017a). Due to the reversibility of the transaminase reaction and the high intracellular glutamate concentrations (Bennett et al., 2009), the availability of an effective OHB reductase with high affinity toward the synthetic substrate and high overall catalytic efficiency is crucial to shift the *in vivo* pathway flux in the direction of the target product DHB. Thus, further engineering might be required to reduce the K_m_ of the engineered OHB reductase variant.

Several studies showed how increased NADPH availability resulted in higher yields and productivities of NADPH-dependent product formations. To show the full potential of our engineered NADPH-dependent OHB reductase, we engineered the host strain toward increased NADPH availability using common strategies, including chromosomal deletion of *Ec.pfkA* to increase the flux through the PPP, deletion of *Ec.sthA*, and chromosomal overexpression of *Ec.pntAB* (Kabus et al., 2007; Rathnasingh et al., 2012; Shi et al., 2013; Cui et al., 2014; Cabulong et al., 2019; Hao et al., 2020; Chen et al., 2024; Zhang et al., 2021; Chin and Cirino, 2011). To investigate the potential effects of increased precursor supply, we also evaluated DHB production in NADPH-overproducing host strains expressing the NADH-dependent OHB reductase *Ec.mdh* ^*5Q*^. Noticeably, we observed a 19% increased DHB yield in our producer strain EcHS5 deleted for *sthA*, expressing *Ec.alaC*_*A142P:Y275D*_ and *Ec.mdh* ^*5Q*^ and a 25% yield increase in the strain EcHS6 overexpressing *pntAB* compared to the respective reference strain EcHS3 (0.16 mol ±0.001 mol_DHB_ mol_Glucose_^−1^). These improvements could possibly be ascribed to an enhancement of homoserine production, as two mol of NADPH are necessary to produce one mol of homoserine. However, the positive effect of engineering NADPH supply via *pntAB* overexpression was clearly strongest in the producer strain EcHS11 expressing the engineered NADPH-dependent *Ec.mdh* ^*7Q*^, reaching the highest DHB yield on glucose (0.25 ± 0.01 mol_DHB_ mol_Glucose_^−1^) reported so far. Although much higher DHB yields starting from homoserine have been reported (0.94 mol mol^−1^) with high-cell densities in recent work of Liu et al. (2022), such an approach requires the separate fermentation of L-homoserine from glucose, thereby increasing production costs. Furthermore, to the best of our knowledge, the authors did not report yields taking into account the use of the initial glucose substrate. In this study, we report a 50% increase in DHB yield with producer strain EcHS11, compared to the reference EcHS1 (0.10 ± 0.004 mol_DHB_ mol_Glucose_^−1^). It would be worthwhile investigating whether DHB titers could be increased further through enhanced NADPH availability. In a previous study, Ng et al. (2015) presented a promising strategy to increase the NADPH regeneration rate in *E. coli* by 25-fold, based on the heterologous expression of a synthetic Entner–Doudoroff pathway.

In a larger, more general context, our study shows how streamlining NADPH cofactor preference of the biosynthetic pathway (by enzyme engineering) and NADPH cofactor supply (by metabolic engineering) can increase the efficiency of aerobic biosyntheses of reduced compounds.

## Data Availability

The original contributions presented in the study are included in the article/Supplementary Material; further inquiries can be directed to the corresponding author.
