# Supplementary material for: Cofactor engineering for improved production of 2,4-dihydroxybutyric acid via the synthetic homoserine pathway
Source: Front Bioeng Biotechnol. 2025 Feb 20;13:1504785. doi: 10.3389/fbioe.2025.1504785 (PMC11882521; doi:10.3389/fbioe.2025.1504785)
Supplement: Supplementary file 1 [file DataSheet1.docx]

Supplementary Material

**Cofactor engineering for improved production of** **2,4-dihydroxybutyric acid via the synthetic homoserine pathway**

**Nadine Ihle^1^, Laura Grüßner^1^, Ceren Alkim^2^, T.A. Stefanie Nguyen^1^, Thomas Walther^1^, Cláudio J.R. Frazão^1*^**

^1^Chair of Bioprocess Engineering, Institute of Natural Materials Technology, TU Dresden, Bergstraße 120, 01062 Dresden, Germany

^2^Toulouse Biotechnology Institute, UMR INSA-CNRS5504 and UMR INSA-INRAE 792; 135 avenue de Rangueil, 31077 Toulouse, France

***Correspondence:**

Cláudio J.R. Frazão

Email: [claudio.frazao@tu-dresden.de](mailto:claudio.frazao@tu-dresden.de)

**Contents summary:**

Table S 1 | Primers used in this study.

Figure S 1 | Multiple protein sequence alignment of NAD(H)-dependent malate dehydrogenases (Mdh), NAD(H)-dependent lactate dehydrogenases (Ldh) and NADP(H)-dependent Mdhs.

Figure S 2 | Specific activity of Ec.Mdh^5Q^ and Ec.Mdh^7Q^ in dependence of NADH (A, B), NADPH (C, D) and OHB (E, F) concentration.

Table S 1 | Primers used in this study. 5’ Overhang sequences are underlined, Mutations are marked by grey background, RBS sequences are marked italic, restriction enzyme recognition sites are marked bold in respective primers.

| **Ref.** | **Sequence 5’ 🡪 3’** | **Application** |
| --- | --- | --- |
| **Primers for site-directed mutagenesis (SDM)** | | |
| TW261 | TCAGAACTCTCTCTGTATGGCATCGCTCCAGTGACTCCCGG | SDM *Ec-mdh_D34G_* (fwd) |
| TW262 | CCGGGAGTCACTGGAGCGATGCCATACAGAGAGAGTTCTGA | SDM *Ec-mdh_D34G_* (rev) |
| TW263 | GAACTCTCTCTGTATGATTCTGCTCCAGTGACTCCCGGTG | SDM *Ec-mdh_I35S_* (fwd) |
| TW264 | CACCGGGAGTCACTGGAGCAGAATCATACAGAGAGAGTTC | SDM *Ec-mdh_I35S_* (rev) |
| TW558 | GAACTCTCTCTGTATGGCAAAGCTCCAGTGACTCCCGGTG | SDM *Ec-mdh_D34G:I35K_* (fwd) |
| TW559 | CACCGGGAGTCACTGGAGCTTTGCCATACAGAGAGAGTTC | SDM *Ec-mdh_D34G:I35K_* (rev) |
| TW560 | GAACTCTCTCTGTATGGCCGTGCTCCAGTGACTCCCGGTG | SDM *Ec-mdh_D34G:I35R_* (fwd) |
| TW561 | CACCGGGAGTCACTGGAGCACGGCCATACAGAGAGAGTTC | SDM *Ec-mdh_D34G:I35R_* (rev) |
| TW562 | GAACTCTCTCTGTATGGCACCGCTCCAGTGACTCCCGGTG | SDM *Ec-mdh_D34G:I35T_* (fwd) |
| TW563 | CACCGGGAGTCACTGGAGCGGTGCCATACAGAGAGAGTTC | SDM *Ec-mdh_D34G:I35T_* (rev) |
| **Primers for pZA23 plasmid construction** | | |
| TW2949 | TAAGCA**GCGGCCGC***GTTTAACTTTAAGAAGGAGATATACC*ATGAAAGTCGCAGTCCTCG | Amplification of *Ec-mdh^7Q^* (fwd), Restriction site **NotI** |
| TW2950 | TCGTTA**TCTAGA**TTACTTATTAACGAACTCTTCGCCCAG | Amplification of *Ec-mdh^7Q^* (rev), Restriction site **XbaI** |
| **Primers for chromosomal integration of the proD promoter to overexpress *pntAB*** | | |
| TW2426 | CGTTAATATTTTGCGAGTTCACGCCGAAATACTGATTTTTGGCGCTAGATCACAGGCATAGTGTAGGCTGGAGCTGCTTC | Amplification of Kan^R^-ProD cassette for integration at *pntA* locus (fwd) |
| TW2427 | GCAACACGGGTTTCATTGGTTAACCGTTCTCTTGGTATGCCAATTCGCATATAATACCTCCTAAAGTTAAACAAAATTATTTGTAG | Amplification of Kan^R^-ProD cassette for integration at *pntA* locus (rev) |
| **Verification of chromosmal modifications** | | |
| TW1805 | cgggctgtcgttgtttacgc | Verify *metL* deletion (fwd) |
| TW1806 | gctggcgtgaaaaaagctcat | Verify *metL* deletion (rev) |
| TW1807 | atgaaacgcattagcaccac | Verify *thrA* deletion (fwd) |
| TW1808 | ctcccagcactgataaacga | Verify *thrA* deletion (rev) |
| TW2424 | TTGCTCCAGTATTGTGAATGAACG | Verify *sthA* deletion (fwd) |
| TW2425 | GGAAGATGGTCACTGTTTGC | Verify *sthA* deletion (rev) |
| TW2428 | AAACGTGGCTGATTATTGC | Verify chromomal integration of *proD* promoter (fwd) |
| TW2429 | CATCGAGCTTAGTGCGTCC | Verify chromomal integration of *proD* promoter (fwd) |
| TW2512 | cgcggctactttcttcat | Verify *ldhA* deletion (fwd) |
| TW2513 | cggctttatatttacccagcat | Verify *ldhA* deletion (rev) |
| TW2520 | taaacggtctatgctttcataa | Verify *pfkA* deletion (fwd) |
| TW2521 | tgagggattaaaaaggcgg | Verify *pfkA* deletion (rev) |


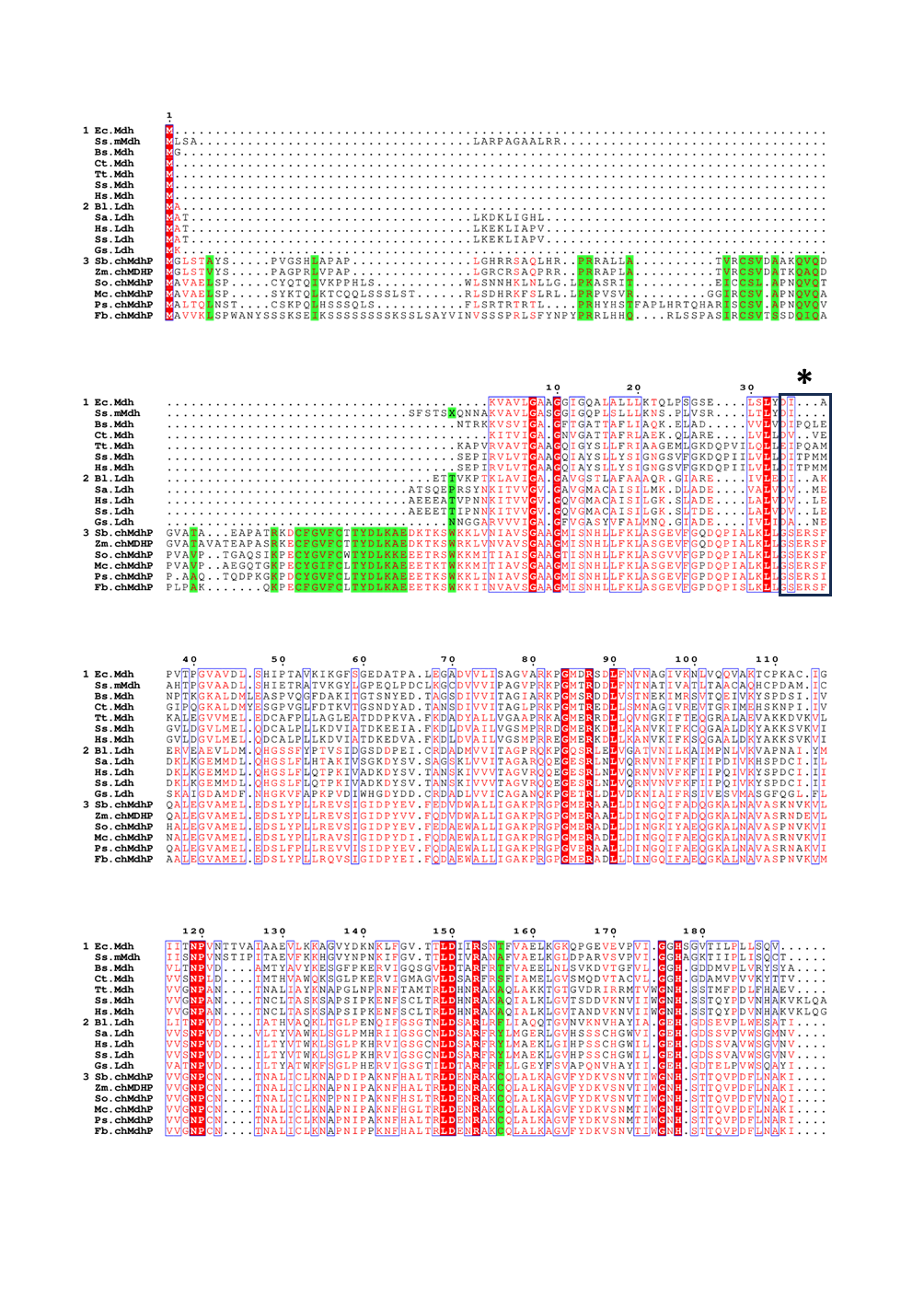


**Figure S 1 | Multiple protein sequence alignment of NAD(H)-dependent malate dehydrogenases (Mdh), NAD(H)-dependent lactate dehydrogenases (Ldh) and NADP(H)-dependent Mdhs.**


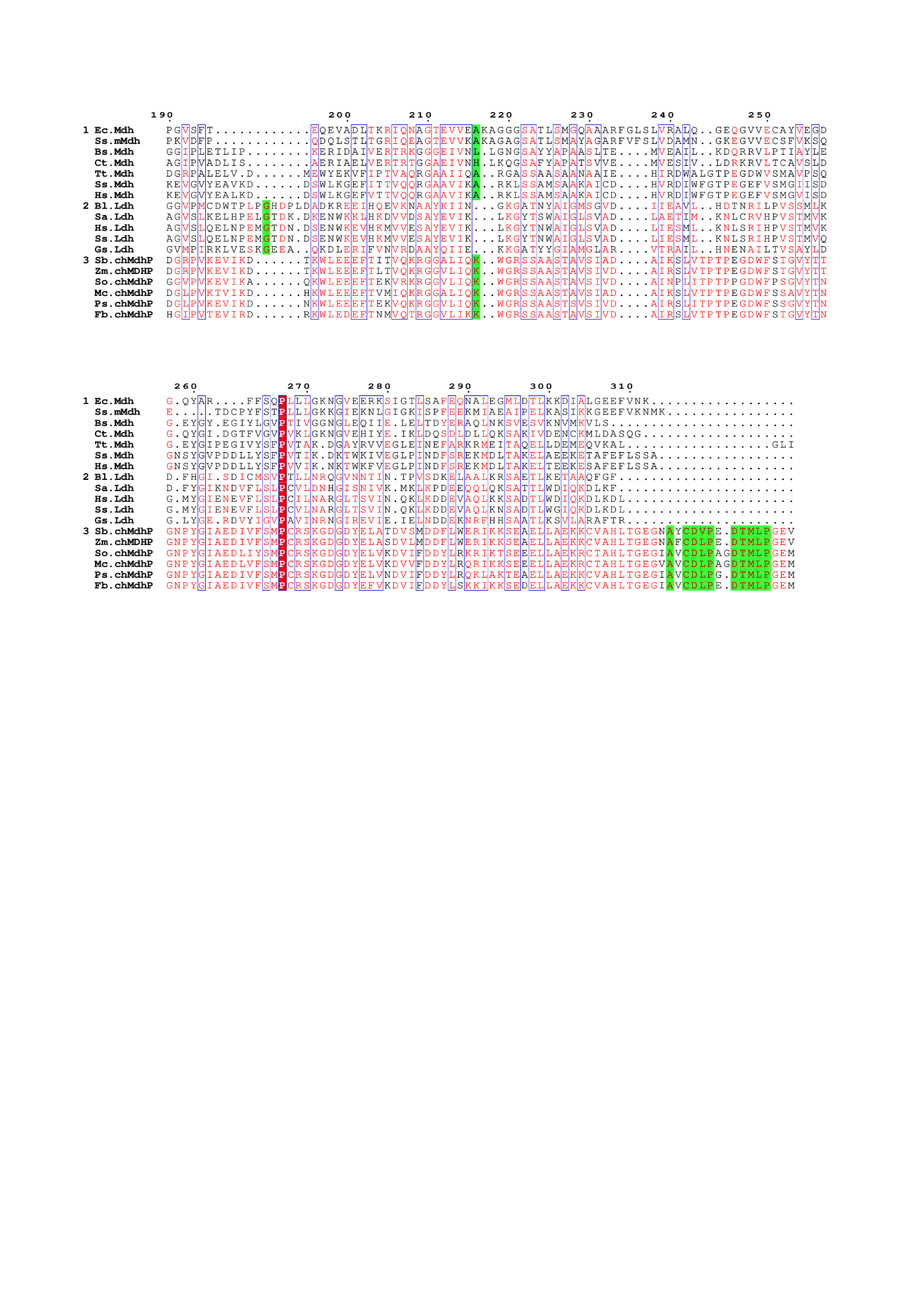


**Figure S 1 continued** | **Multiple protein sequence alignment of NAD(H)-dependent malate dehydrogenases (Mdh), NAD(H)-dependent lactate dehydrogenases (Ldh) and NADP(H)-dependent Mdhs.** The alignment was performed using MAFFT FFT-NS-i v7.525 provided by EMBL-EBI (Madeira et al., 2024). Colors represent sequence similarity score, which was calculated groupwise based on the Risler matrix using ESpript v 3.0 (Robert & Gouet, 2014). The co-factor selectivity control loop corresponding to position 34-38 in Ec.Mdh is highlighted by an asterisk. Uniprot IDs: Group 1 – NAD(H)-dependent Mdhs : Ec.Mdh: [P61889](https://www.uniprot.org/uniprotkb/P61889/entry) (cytosolic), Ss.mMdh: [P00346](https://www.uniprot.org/uniprotkb/P00346/entry) (mitochondrial), Bs.Mdh: [P49814](https://www.uniprot.org/uniprotkb/P49814/entry) (cytosolic), Ct.Mdh: [P80039](https://www.uniprot.org/uniprotkb/P80039/entry) (cytosolic), Tt.Mdh: [P10584](https://www.uniprot.org/uniprotkb/P10584/entry) (cytosolic), Ss.Mdh: [P11708](https://www.uniprot.org/uniprotkb/P11708/entry) (cytosolic), Hs.Mdh: [P40925](https://www.uniprot.org/uniprotkb/P40925/entry) (cytosolic); Group 2 – NAD(H)-dependent Ldhs: Bl.Ldh: [E8ME30](https://www.uniprot.org/uniprotkb/E8ME30/entry), Sa.Ldh: [P00341](https://www.uniprot.org/uniprotkb/P00341/entry), Hs.Ldh: [P07195](https://www.uniprot.org/uniprotkb/P07195/entry), Ss.Ldh: [P00336](https://www.uniprot.org/uniprotkb/P00336/entry), Gs.Ldh: [P00344](https://www.uniprot.org/uniprotkb/P00344/entry); Group 3 – NADP(H)-dependent chloroplast Mdhs (chMdhP): Sb.chMdhP: [P17606](https://www.uniprot.org/uniprotkb/P17606/entry) (chloroplastic), Zm.chMdhP: [P15719](https://www.uniprot.org/uniprotkb/P15719/entry) (chloroplastic), So.chMdhP: [P52426](https://www.uniprot.org/uniprotkb/P52426/entry) (chloroplastic), Mc.chMdhP: [Q05145](https://www.uniprot.org/uniprotkb/Q05145/entry) (chloroplastic), Ps.chMdhP: [P21528](https://www.uniprot.org/uniprotkb/P21528/entry) (chloroplastic), Fb.chMdhP: [P46489](https://www.uniprot.org/uniprotkb/P46489/entry) (chloroplastic). Legend: Red box, white character = strict identity, red character = similarity in a group, blue frame = similarity across groups, green box: differences between conserved groups.


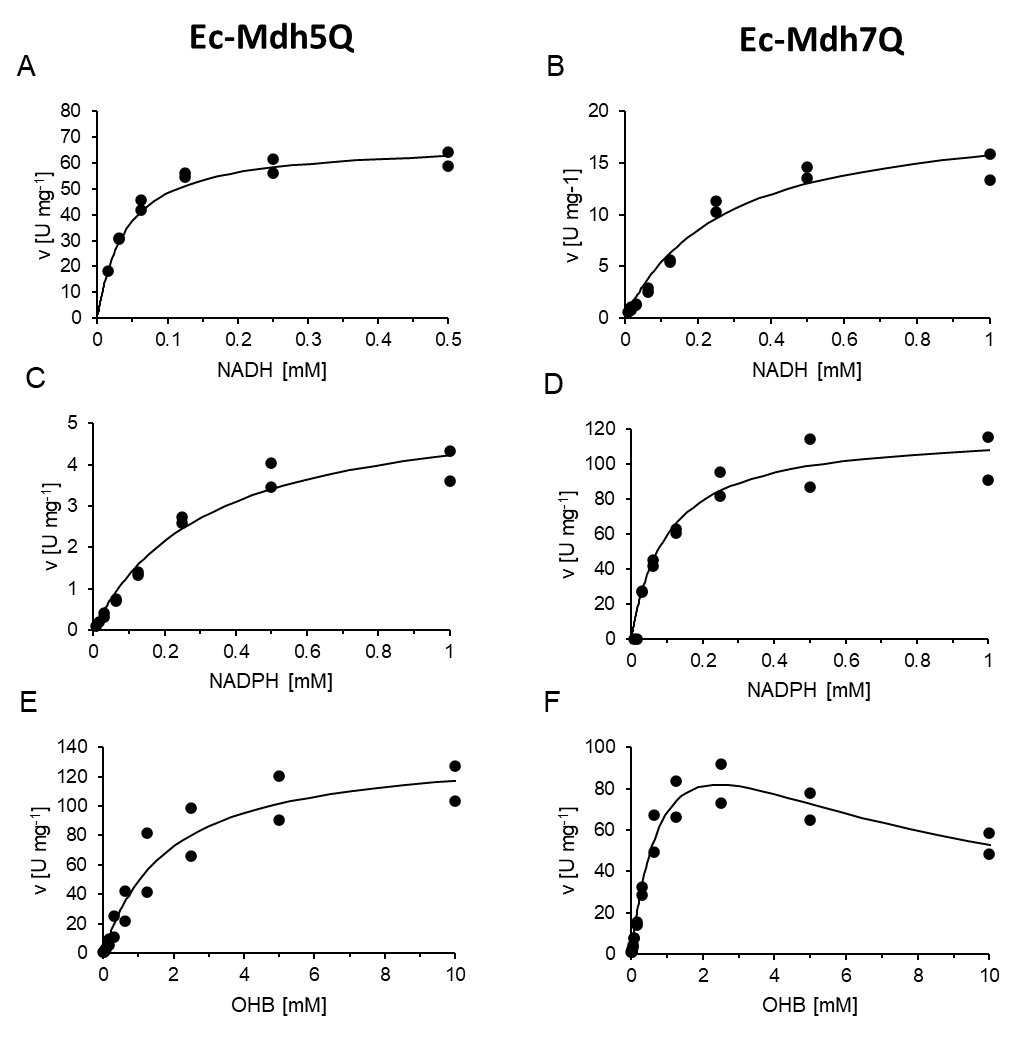


Figure S 2 | Specific activity of Ec.Mdh^5Q^ and Ec.Mdh^7Q^ in dependence of NADH (A, B), NADPH (C, D) and OHB (E, F) concentration. In A-D, specific OHB reductase activities were determined at fixed concentrations of substrate (OHB, 2 mM) and variable concentrations of NAD(P)H (1-0.03 mM). In E, specific OHB reductase activities were determined at fixed concentrations (0.25 mM) of the preferred co-substrate NADH and variable amounts of OHB (10-0.005 mM). Experimental data was fitted to the Michaelis-Menten model. In F, specific OHB reductase activities were determined at fixed amounts (0.25 mM) of the preferred co-substrate NADPH and variable amounts of OHB (10-0.005 mM). Experimental data was fitted to the substrate inhibition model.

**Supplementary references**

Madeira, F., Madhusoodanan, N., Lee, J., Eusebi, A., Niewielska, A., Tivey, A. R. N., Lopez, R., & Butcher, S. (2024). The EMBL-EBI Job Dispatcher sequence analysis tools framework in 2024. *Nucleic Acids Research*, *2024*, 1–5. https://doi.org/10.1093/nar/gkae241
